# New Biomarker in Chagas Disease: Extracellular Vesicles Isolated from Peripheral Blood in Chronic Chagas Disease Patients Modulate the Human Immune Response

**DOI:** 10.1155/2021/6650670

**Published:** 2021-01-11

**Authors:** Rafael Pedro Madeira, Lavínia Maria Dal'Mas Romera, Paula de Cássia Buck, Charles Mady, Barbara Maria Ianni, Ana Claudia Torrecilhas

**Affiliations:** ^1^Disciplina de Infectologia, Departamento de Medicina, Universidade Federal de São Paulo (UNIFESP), São Paulo, Brazil; ^2^Laboratório de Imunologia Celular e Bioquímica de Fungos e Protozoários, Departamento de Ciências Farmacêuticas, Universidade Federal de São Paulo (UNIFESP), Diadema, Brazil; ^3^Unidade Clínica de Miocardiopatias, Instituto do Coração, Universidade de São Paulo (USP), São Paulo, Brazil

## Abstract

Chagas disease, a neglected tropical disease (NTD) caused by the flagellated protozoan *Trypanosoma cruzi* (*T. cruzi*), is a major public health problem. It was initially restricted to Latin America, but it is now expanding globally. Host and pathogen interactions are crucial in the establishment of disease, and since 1970, it has been known that eukaryotic cells release extracellular vesicles (EVs), which in turn have an important role in intercellular communication in physiological and pathological conditions. Our study proposed to characterize and compare circulating EVs isolated from the plasma of chronic Chagas disease (CCD) patients and controls. For this, peripheral blood was collected from patients and controls, and mononuclear cells (PBMCs) were isolated and stimulated with parasite EVs, showing that patient cells released fewer EVs than control cells. Then, after plasma separation followed by EV total shedding enrichment, the samples were subjected to ultracentrifugation to isolate the circulating EVs, which then had their size and concentration characterized by nanoparticle tracking analysis (NTA). This showed that patients had a lower concentration of circulating EVs while there were no differences in size, corroborating the *in vitro* data. Additionally, circulating EVs were incubated with THP-1 cells (macrophages) that, after the interaction, had their supernatant analyzed by ELISA for cytokine detection. In relation to their ability to induce cytokine production, the CCD patient EVs were able to induce a differential production of IFN-*γ* and IL-17 in relation to controls, with differences being more evident in earlier/less severe stages of the disease. In summary, a decreased concentration of circulating EVs associated with differential activation of the immunological system in patients with CCD is related to parasite persistence and the establishment of chronic disease. It is also a potential biomarker for monitoring disease progression.

## 1. Introduction

*Trypanosoma cruzi* is a protozoan parasite and the causative agent of Chagas disease (CD), also called American trypanosomiasis. CD is a systemic and chronic disease that is considered one of the 13 most Neglected Tropical Diseases (NTD) worldwide by the World Health Organization [1]. These diseases persist exclusively in the poorest and most marginalized populations, living without adequate sanitation and in close contact with infected vectors and reservoirs. The disease affects 8 million people in Latin America from Mexico to Argentina, and there is a potential public health problem in the USA, as well as Europe and Asia, due to increasing immigration from endemic countries [2, 3].

In its chronic phase, the clinical presentations range from the absence of signs and symptoms (the indeterminate form) to a severe cardiac, digestive, or cardiodigestive burden with high morbidity and mortality [3–5]. The gold standard for CCD diagnosis is a combination of two different serological assays, enzyme-linked immunosorbent assays (ELISA), hemagglutination inhibition assays (HAI), or indirect immunofluorescence (IIF), with the addition of a third test if the first two have discrepant results [6]. This combination approach is due to the reduced specificity of the tests based on the type of antigen used, which may lead to cross-reactivity with other parasitic diseases such as leishmaniasis [7]. There are still no available methodologies for the prognosis of confirmed chronic patients, although some studies using molecular and imaging approaches have shown some degree of correlation with the disease severity [8–10].

Once referred to as “platelet dust” by Wolf in 1967, extracellular vesicles (EVs) have now been extensively studied due to their role in intercellular communication and physiological and pathological conditions [11–13]. Their role in assessing disease progression as well as in establishing a prognosis suggests a potential use for them as biomarkers in noncommunicable diseases, such as cancer, but also in infectious diseases such as latent tuberculosis infection [14–16].

In a previous work by our group, we showed that *T. cruzi* trypomastigotes derived from infected mammalian cells released vesicles into the medium and that EVs of different sizes were associated with both the parasite membrane and the culture medium ([17]; [18]). These EVs carry glycoproteins are responsible for cell activation via TLR2, and it modulates the host innate immune response and increases the number of cell infections and intracellular parasites [19, 20]. The major glycoproteins from parasite surface, such as TS/gp85 glycoproteins and mucins, were found in EVs release by infective trypomastigote forms of *T. cruzi*. The mucins are the major surface glycoproteins from *T. cruzi* cell surface and are rich in O-linked *α*-galactosyl (*α*Gal) epitope-containing oligosaccharides [21]. These *α*-Gal epitopes are the major target of lytic anti-*α*Gal antibodies, which are the predominant IgG during Chagas' disease and have the ability to control parasitic infection [21]. Furthermore, the addition of sialic acid residues confers a protection to the parasite against the anti-*α*Gal antibodies [22]. In fact, a proteomic analysis of this EVs isolated from trypomastigotes forms show that about 60% of the hits correspond to proteins of the 85 K Daltons family (gp85/TS) and mucins of the protozoa parasite [20]. Those glycoproteins are involved in the parasite host interaction and invasion by the parasite ([23–27]; [18, 28]). EVs contain virulence factors involved in pathogenesis and immunopathology, suggesting their ability to modulate host immune responses and inflammation.

*In vivo*, EVs increase the number of amastigote nests in heart tissue and carry virulence factors that are important for pathogenesis ([18–20, 29]).

The applications of EVs in clinical therapy have rapidly advanced in the past decade. The main challenges in clinical investigation are to promote the use of EVs in clinical trials and during the follow-up of many inflammatory and infectious diseases, as there is still no biomarker for infectious disease progression. Since EVs were demonstrated to be important for the development of heart parasitism and inflammation in animal models of infection, we proposed to characterize the peripheral blood circulating population of EVs in CCD patients as well as their immunomodulatory capacity *in vitro.*

## 2. Materials and Methods

### 2.1. Ethics Statement

All of the experiments in this work were approved by the Federal University of São Paulo Ethics Committee in Research, CEP/UNIFESP (CAAE: 70749317.2.0000.5505), and samples from both patient and healthy controls were only collected after individuals agreed to participate and signing a written informed consent form.

### 2.2. Participants

The study included 70 individuals, 40 chronic Chagas disease patients and 30 healthy controls, selected from two university outpatient clinics (infected) and laboratory staff (noninfected) between January 2019 and May 2019. All patients, despite being in different disease stages, had a positive serological diagnosis for Chagas disease using ELISA and IIF. The patients were further divided into groups according to disease stage, degree of cardiac burden, and functional classification by New York Heart Association parameters as described in Table 1.

### 2.3. Obtaining and Isolating EVs Released by Trypanosoma Cruzi

*T. cruzi* culture (Y strain) was maintained by infection of green monkey kidney LLC-MK2 epithelial cells (ATCC, Manassas, VA) in Dulbecco's modified eagle (DME) medium supplemented with 10% fetal bovine serum (FBS) at 37°C, under 5% CO_2_ atmosphere, as described elsewhere [29]. Total *T. cruzi* shed vesicles were obtained from the culture medium supernatant of tissue culture cell-derived trypomastigotes (TCTs), which were harvested 5 to 9 days after the infection of LLC-MK2 cells. Parasites were counted, centrifuged (15 min, 1,500-2,000g, 10°C), and resuspended in DME medium supplemented with 5% FBS, at a concentration of 1 × 10^9^ parasites/mL of medium. After incubation for 2-3 h at 37°C, under 5% CO_2_ atmosphere, trypomastigotes were removed by centrifugation (10 min, 3000 g, 10°C), and the supernatant containing the total shed material was filtered through a 0.45 *μ*m cartridge [20, 29].

### 2.4. Fractionation of T. cruzi Vesicles

The total shed material was 2-fold diluted with 200 mM ammonium acetate (pH 6.5) and loaded onto a Sepharose CL-4B column (1 × 40 cm, GE Healthcare, Piscataway, NJ) preequilibrated with 100 mM ammonium acetate (pH 6.5). The column was eluted with the equilibration buffer, in a flow rate of 0.2 mL/min using a peristaltic pump (GE Healthcare). Fractions (*N* = 80) of 1 mL were collected and then screened by chemiluminescent enzyme-linked immunosorbent assay (CL-ELISA) as described elsewhere [20], using anti-*T. cruzi* membrane polyclonal antibody (mouse) or anti-Alpha Gal purified from sera of chronic Chagasic patients (human Ch anti-*α*Gal), as described [21]. The most reactive fractions being pooled and concentrated in a vacuum centrifuge and then resuspended in filtered PBS for further analysis by nanoparticle tracking analysis (NTA) as previously described [20, 29, 30].

### 2.5. Blood Collection and Sample Preparation

Blood from the CCD samples was collected in lithium heparin for peripheral blood mononuclear cell (PBMC) isolation in a Ficoll-Paque (GE Healthcare) density gradient according to the manufacturer's instructions. Additionally, blood collected in sodium EDTA tubes was left at ambient temperature for 4 h and then incubated overnight at 4°C for plasma separation, which was used as the starting material for the isolation of circulating EVs.

### 2.6. Isolation of EVs from CCD Patient Plasma

After plasma separation from the blood, it was submitted to centrifugation at 100,000g for 1 h in a Thermo Scientific™ Sorvall™ WX100 Ultra Centrifuge using a fixed angle rotor (Thermo Scientific™ T-8100 Fixed Angle Rotor). The pellets were resuspended in filtered PBS (0.2 *μ*m syringe filter) and then analyzed by NTA.

### 2.7. Scanning Electron Microscopy (SEM)

PBMCs, after incubation for 24 and 48 h with and without EVs isolated from parasites, were fixed in a 2.5% glutaraldehyde solution, postfixed with osmium tetroxide, treated with tannic acid, and dehydrated with ethanol [29]. The samples were observed in a field emission FEI Quanta 250 FEG scanning electron microscope (FEI, OR, USA).

### 2.8. PBMC–Parasite EV Interaction Assay

Following isolation, 1 × 10^5^ PBMCs were seeded on 24-well plates and incubated for 24 h in culture medium. After 24 h, the cells were washed with PBS and then incubated with parasite EVs at a 1 : 100 (cell : EV) ratio, parasite extract (obtained from freeze-thawing and filtering an equivalent of 10^8^ parasites), and culture medium for another 24 h at 37°C and 5% CO_2_. Supernatants were centrifuged for 10 minutes at 750g and then analyzed by NTA.

### 2.9. Nanoparticle Tracking Analysis (NTA)

EVs isolated from parasites and samples from patients were diluted in filtered PBS and then loaded into the NanoSight NS300 equipment (Malvern Panalytical) coupled to an sCMOS camera at a 532 nm wavelength, camera level set to auto, threshold and focus set manually to optimize readings as per the manufacturers' instructions. Readings were taken in triplicate for 30 seconds at 25 frames per second, and the data were analyzed using Nanoparticle Tracking Analysis software (NTA version 3.2 Dev Build 3.2.16).

### 2.10. Immunological Assays

THP-1 cells (ATCC® TIB-202™ Cell Type: monocyte) (10^7^ cells) were differentiated with macrophages. Fifty ng/mL phorbol 12-myristate 13-acetate was primed with 10 ng/mL human recombinant interferon-gamma (IFN-*γ*, GenScript). The cells were then incubated with EVs isolated from plasma patients in a 1 : 100 (cell : EV) ratio. All incubations were performed at 37°C in 5% CO_2_ for 24 h. Supernatants were collected for cytokine assays. Culture medium alone was used as a negative control.

### 2.11. Cytokine Measurements

For the ELISA cytokine detection, supernatants were collected, and cytokines were determined using Human Cytokine assay kits (Human DuoSet ELISA, R&D Systems) according to the manufacturer's specifications. The cytokines TNF-*α*, IFN-*γ*, IL-4, IL-5, IL-6, IL-10, IL12p70, and IL-17 were assessed and, when detected in the supernatant, had their concentration measured and compared to controls and between subgroups of patients.

### 2.12. Statistical Analysis

All data sets were assessed using GraphPad Prism 7.0 software (GraphPad Software Inc., San Diego, USA) and Orange (University of Ljubljana, Slovenia). As appropriate, Spearman's correlation, unpaired *t*-tests with Welch's correction, and ordinary one-way ANOVA followed by Dunnett's multiple comparisons test were performed, and the results are presented as the means ± 95% confidence intervals.

## 3. Results

### 3.1. Patient Distribution among Subgroups and Analysis of Clinical Data

**Figure 1:**
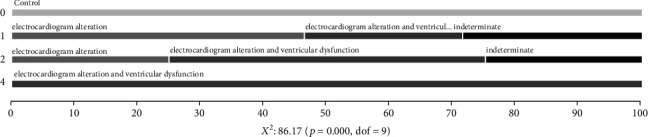
CCD patients' clinical data analysis. Frequency of patients by the stage of cardiac burden in relation to the NYHA functional classification.

Our cohort of patients was evenly distributed between male and female individuals as well as disease stage and degree of cardiac burden, with the majority (39 out of 40) of patients being over 40 years old and in NYHA class I (Table 1). Additionally, there was a correlation between a higher degree of cardiac burden and NYHA functional classification (Figure 1). Full clinical data from the patients are presented in Supplementary Table 1.

### 3.2. PBMC Purification from Chronic Chagas Disease Patients Releases Fewer EVs Than from Healthy Individuals

**Figure 2:**
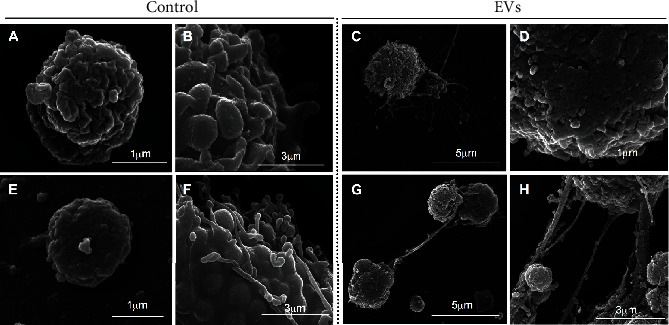
Scanning electron microscopy (SEM) of CCD PBMCs releasing EVs after 24 and 48 h of parasite EV stimulus. 24 h control in (a, b), 48 h control in (e, f), 24 h parasite EV stimulus in (c, d), and 48 h parasite EV stimulus in (g, h).

**Figure 3:**
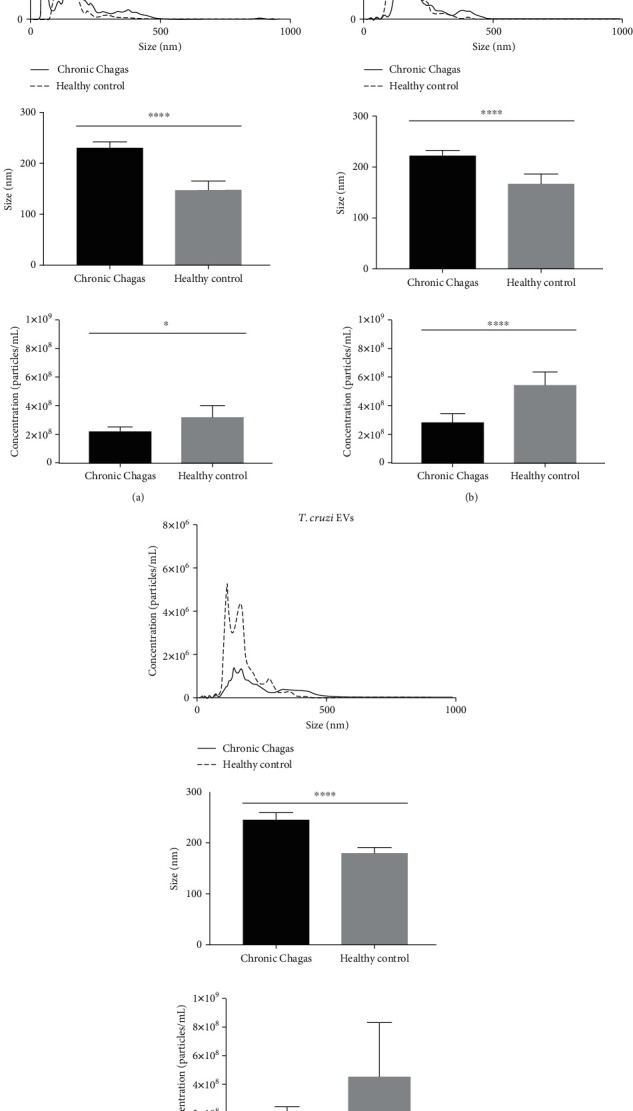
Comparison of EV profiles from CCD and CTRL PBMCs after a 24 h incubation. Concentration/size distribution (top), mean size ± 95%CI (center) and mean concentration ± 95%CI (bottom) after (a) culture medium (^∗^*p* = 0.0326 and ^∗∗∗∗^*p* < 0.0001), (b) *T. cruzi* extract (^∗∗∗^*p* = 0.0003 and ^∗∗∗∗^*p* = 0.0002), or (c) *T. cruzi* EV stimuli (^∗∗∗∗^*p* < 0.0001).

PBMCs isolated from chronic Chagas disease patients' blood can constitutively release EVs, as shown by scanning electron microscopy (Figure 2). When stimulated with *T. cruzi* extract or *T. cruzi* EVs, PBMC EVs presented a similar size dispersion but a smaller mean particle size than healthy individuals (Figure 3, top and center). For their mean concentration, even though they were larger in size, they were fewer in quantity when compared to healthy individuals (Figure 3, bottom).

### 3.3. Chronic Chagas Disease Patients Have Fewer Total Circulating EVs in Plasma Than Healthy Individuals (Control)

**Figure 4:**
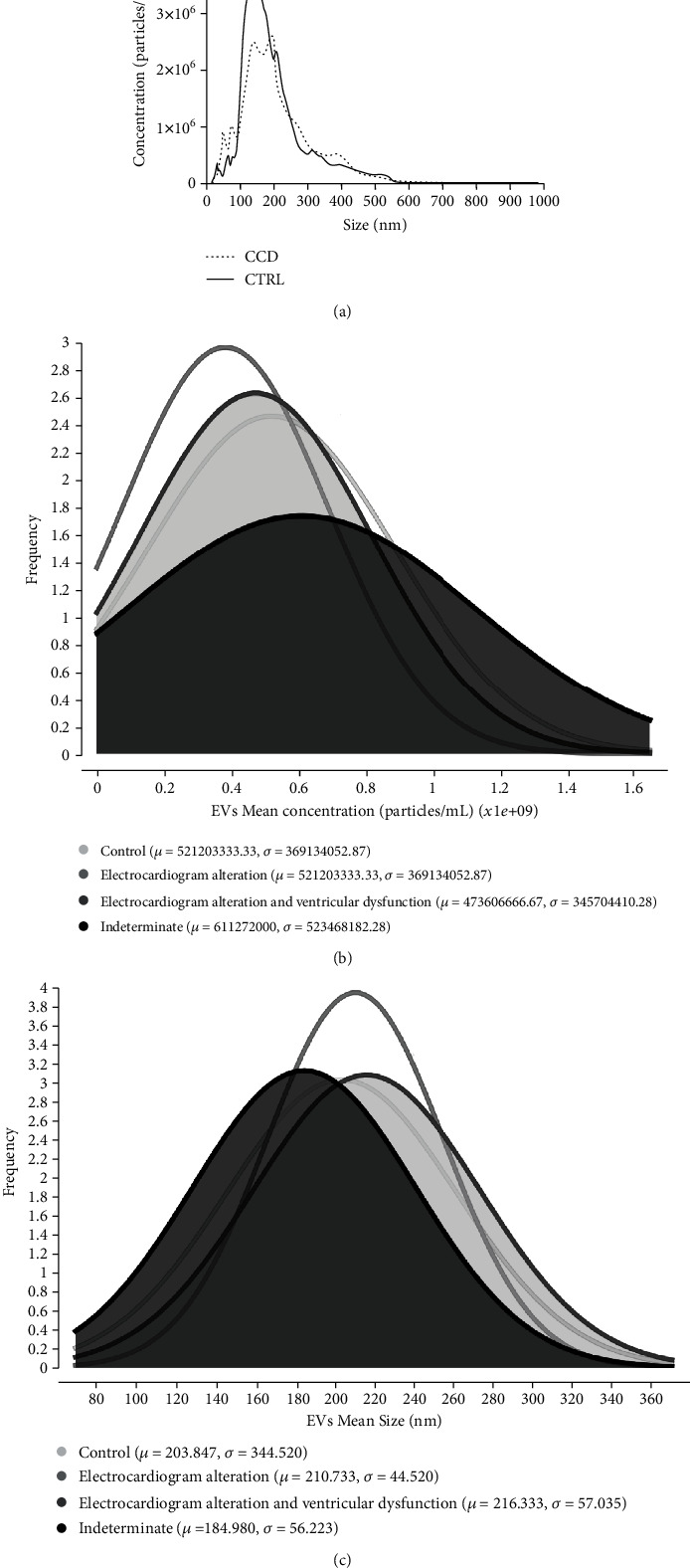
Comparison of circulating EV profiles in CCD patients in relation to CTRL. (a) Concentration (particles/mL) × size (nm) profile. (b) Frequency of EV concentration among different degrees of cardiac burden. (c) Frequency of EV size among different stages of cardiac burden.

After isolation from plasma, nanoparticle tracking analysis revealed that patients with chronic Chagas disease had a lower concentration of total circulating EVs when compared to healthy individuals, corroborating the observed results from peripheral blood mononuclear cells (Figure 4(a)). When analyzing the frequencies of the concentration values throughout subgroups of patients, it was observed that reduced concentrations of circulating EVs were associated with alterations in cardiac clinical parameters (Figure 4(b)), but this phenomenon was not observed when assessing EV size (Figure 4(c)).

### 3.4. EVs from Chronic Chagas Disease Patients Induce Differential Cytokine Production and Release from THP-1 Cells (Macrophages)

**Figure 5:**
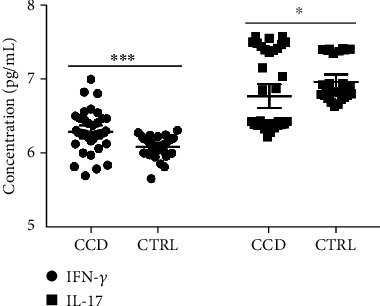
Cytokine production by THP-1 cells (macrophages) quantified in the supernatant by ELISA after 24 h of stimulation with CCD or CTRL EVs. (^∗^*p* = 0.0438 and ^∗∗∗^*p* = 0.0002).

**Figure 6:**
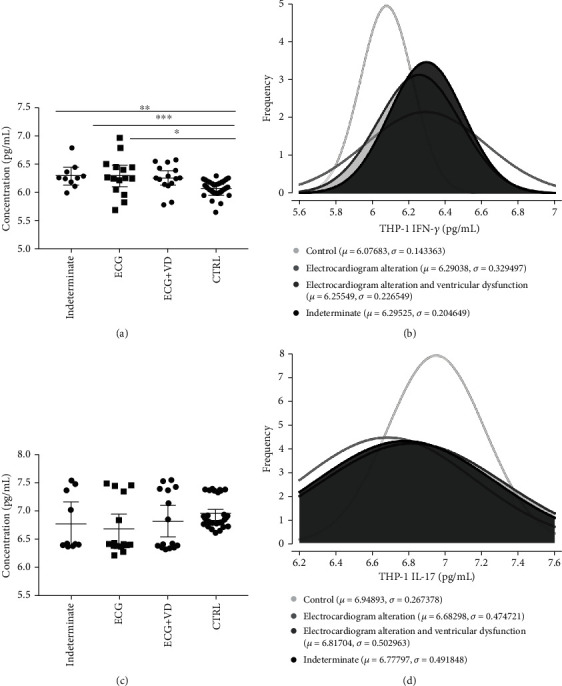
Cytokine production by THP-1 cells (macrophages) quantified in the supernatant by ELISA after 24 h of stimulation with CCD or CTRL EVs among patients grouped based on the clinical stage of the cardiac burden and controls. (a) IFN-*γ* concentration in pg/mL (^∗^*p* = 0.0420, ^∗∗^*p* = 0.0291, and ^∗∗∗^*p* = 0.0118). (b) Frequency of IFN-*γ* concentration values. (c) IL-17 concentration in pg/mL. (d) Frequency of IL-17 concentration values.

**Figure 7:**
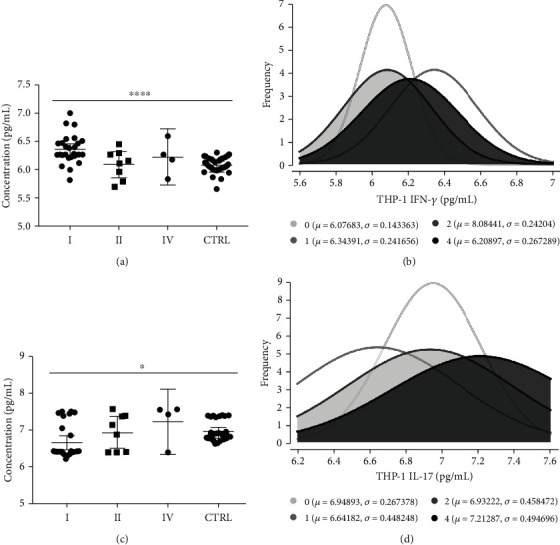
Cytokine production by THP-1 cells (macrophages) quantified in the supernatant by ELISA after 24 h of stimulation with CCD or CTRL EVs among patients grouped based on NYHA functional classification and controls. (a) IFN-*γ* concentration in pg/mL (^∗∗∗∗^*p* = 0.0001). (b) Frequency of IFN-*γ* concentration values (0: CTRL). (c) IL-17 concentration in pg/mL (^∗^*p* = 0.0125). (d) Frequency of IL-17 concentration values (0: CTRL).

**Figure 8:**
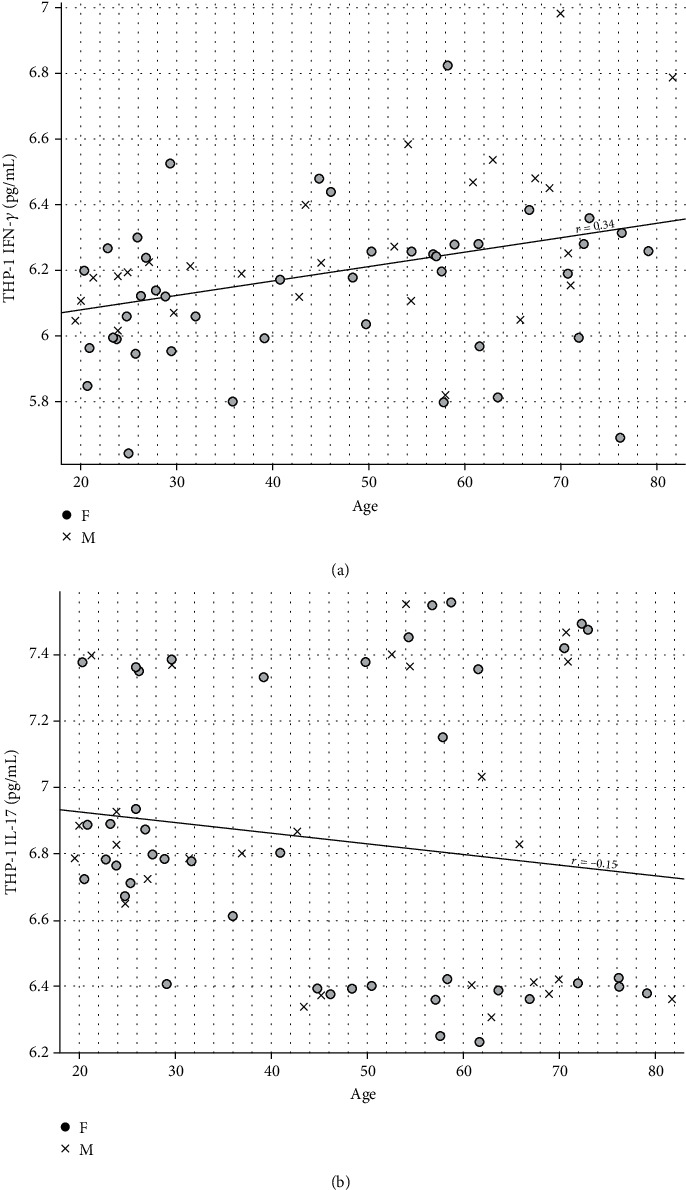
Correlation of age (*X* axis), cytokine production by THP-1 cells (macrophages) quantified in the supernatant by ELISA after 24 h of stimulation with CCD or CTRL EVs (*Y* axis) and sex (•: female; X: male). (a) IFN-*γ* (*r* = 0.34). (b) IL-17 (*r* = −0.15).

After EV stimulation, differentiated and activated THP-1 cells (macrophages) exhibited differential production and release of cytokines in the supernatant. In general, cells that interacted with chronic Chagas patient EVs exhibited a higher production of IFN-*γ* but a lower production of IL-17 (Figure 5). When the data were analyzed based on the different patient subgroups, IFN-*γ* differential production was maintained throughout every subset analyzed, while IL-17 only presented a tendency toward a reduction in patient samples. Taking into consideration the clinical stage of cardiac burden, IFN-*γ* production is higher due to interaction with EVs from patients with the indeterminate form and decreases as the severity of cardiac burden increases (Figure 6(a)), a phenomenon that is clearly observed when the frequencies of each subgroup are plotted against the IFN-*γ* concentration (Figure 6(b)). On the other hand, IL-17 production after EV stimuli showed no significant difference, with only a tendency toward lower levels of this cytokine in the patients (Figures 6(c) and 6(d)). When the data were analyzed based on the New York Heart Association (NYHA) Functional Classification, only EVs from patients included in Class I induced higher IFN-*γ* production (Figure 7(a)) and lower IL-17 production (Figure 7(c)) than the control EVs, as was also seen on the overlapping curves of frequency by cytokine concentration (Figures 7(b) and 7(d), respectively). In addition to the clinical parameters, age showed a positive correlation with IFN-*γ* production (Figure 8(a)) and a negative correlation with IL-17 (Figure 8(b)), but in neither case was sex relevant to the results obtained.

## 4. Discussion

Extracellular vesicles are released from a wide array of cells ranging from prokaryotic organisms to higher eukaryotes and are found in virtually all body fluids in humans [13, 31, 32]. They have important roles in intercellular signaling during physiological processes, such as in kidney physiology, where urinary EVs may play a role in the renin-angiotensin system by carrying angiotensin-converting enzyme and being able to interact with cells in the renal tubule lumen [33]. Another important example of EVs helping to maintain homeostasis is in modulating chemotaxis, signaling, and the proliferation of hematopoietic cells by platelet-derived microparticles [34].

Due to the many different cells circulating in blood, we first wanted to assess whether the mononuclear cell EV-releasing behavior in chronic Chagas disease patients was compatible with what was observed in healthy individuals. Peripheral blood mononuclear cells from chronic Chagas patients released a lower quantity of EVs than cells from healthy individuals when cultivated *in vitro*, and this phenomenon was also observed when quantifying EVs directly from peripheral blood plasma.

The alteration of body fluid EV concentrations in pathological states has been described in both infectious and inflammatory models [35, 36]. In patients with periodontitis, there is a higher concentration of EVs in gingival crevicular fluid, which correlates with the clinical inflammatory periodontal parameters [37]. A similar phenomenon is observed in patients with human African trypanosomiasis, where late stage patients have a higher concentration of EVs in cerebrospinal fluid when compared to early and intermediate stages, with these EVs also showing different functional properties such as altering astrocyte protein expression *in vitro* [38]. In our study, as the EV concentration in patients was lower than that in controls, we hypothesized that this might have led to a loss of function in the human immune response, which in turn contributed to infection persistence and severity, as in a previously described *in vitro* model of *Pseudomonas aeruginosa* infection where the infected cells released fewer EVs that, in turn, carried less CCL4 mRNA, contributing to a less effective immune response [39].

To assess the impact of circulating EVs on the human immune response, despite their decreased number in chronic Chagas patients, we incubated macrophage (THP-1) cells, which were previously differentiated and activated, with EVs and quantified an array of cytokines in the culture supernatant. The importance of studying cytokines in Chagas disease can be exemplified by polymorphisms in genes related to Th1-type T cell differentiation playing a role in genetic susceptibility to chronic Chagas cardiomyopathy [40]. In our model, we observed that while most cytokines analyzed could not be detected or showed no differences among the groups, both IFN-*γ* and IL-17 presented a differential profile when comparing chronic Chagas patients and healthy controls.

We observed that in samples from patients, circulating EVs induced a higher production of IFN-*γ*, corroborating data available from an in vivo chronic model of benznidazole treatments where the treated mice had fewer IFN-*γ*-producing cells as well as an improvement in electrocardiographic alterations. Additionally, circulating IFN-*γ* was positively correlated with the cardiac inflammatory process and parasite burden [41, 42]. In contrast to IFN-*γ*, IL-17 production was diminished after stimulation with patient EVs, which in murine models is associated with compromised parasite control and a reduction of the response magnitude and survival of CD8^+^ T cells [43]. This combination of augmented IFN-*γ* and reduced IL-17 may play an important role in parasite persistence in chronic disease as well as tissue damage in target organs due to continuous inflammatory signaling.

In an attempt to evaluate whether the alterations in circulating EV concentration and subsequent immune activation would be associated with Chagas disease chronification and progression, we stratified our cohort of patients by sex, age, degree of cardiac burden, and functional classification.

Sex can be an important factor in inflammation pathophysiology. In athletes who suffered a concussion, while men have a positive correlation of IFN-*γ* levels with the severity of their symptoms, women have a negative correlation of IFN-*γ* levels and symptom severity [44]. However, apart from a slight difference in healthy individuals, sex was not a factor that could interfere with IFN-*γ* production after stimulus with EVs from chronic Chagas disease patients.

While sex represented no interfering factor with IFN-*γ* production, age proved itself a much more complex factor. During aging, a process called immunosenescence takes place and it is characterized by a decrease in the acute inflammatory response combined with a persistent low-grade inflammatory profile that may lead to a higher risk of infection development as well as participate in the pathogenesis of chronic noncommunicable diseases such as osteoporosis, rheumatoid arthritis, and coronary heart disease [45–47]. After incubation with circulating EVs from patients, macrophages produced more IFN-*γ* than healthy controls. Another point to take into consideration is that almost all of the patients were older than the controls, so a combination of both age and infection might be responsible for the increase in IFN-*γ* levels and the establishment of a basal proinflammatory environment, which in turn could be related to cardiac tissue damage characteristic of chronic symptomatic Chagas disease [48, 49].

IFN-*γ* production represents a major factor in Chagas disease pathogenesis. When we compared different degrees of cardiac damage, clinically assessed by electrocardiography (ECG), we observed that only EVs from patients in the indeterminate stage or with only ECG alterations were able to induce IFN-*γ* production, as EVs from patients with ECG alterations combined with ventricular dysfunction could not. This might suggest that an increase in IFN-*γ* production, and consequently more inflammation, is crucial in the establishment of chronic disease more than in the final stages, where the severity of symptoms is more due to a loss of organ function [50, 51].

In addition to the cardiac burden, the overall effects on its function could also be related to immunological EV-mediated signaling [52, 53]. Corroborating the data derived from patients grouped by ECG, when we looked at the loss of cardiac function, we observed that IFN-*γ* production was also increased when using EVs from patients with no loss of function, validating our hypothesis that a proinflammatory environment is a key point in the establishment of chronic disease. Another important point is that in functionally normal patients, their EVs also led to a decrease in IL-17. A follow-up study in school-aged children with Chagas disease found that higher IL-17A levels were associated with the persistence of infection after treatment with benznidazole, suggesting this cytokine could be a possible biomarker for nonresponse to treatment and the persistence of infection [54]. However, when we analyzed our data under this hypothesis, in contrast to what was previously described, EVs from chronic patients with no cardiac function loss induced a lower production of IL-17, suggesting that in patients who did not receive benznidazole treatment, this cytokine might have another role, even a protective one.

The observed important role of EVs in Chagas disease pathogenesis and chronic disease combined with their altered quantity when compared to healthy individuals suggests EVs are a possible biomarker for disease progression. Even though other situations may alter the concentration of circulating EVs, their differential effect on target cells suggests a composition unlike that seen for healthy individuals' vesicles. Proteomics studies using primary murine or immortalized human cells infected with parasites such as *Plasmodium yoelii* and *Trypanosoma cruzi* demonstrated that EVs released from infected cells carry parasite molecules as well their own cargo, but none showed this phenomenon using circulating EVs or EVs from patients [55, 56].

In the case of Chagas disease, some highly expressed parasite molecules are important for infection, such as the virulence factors *trans*-sialidase and cruzipain [57]. These molecules are able to induce a humoral immune response that can be detected and used for diagnostics and treatment monitoring, even though cross-reactivity with other infections also exists [57–59]. Considering the demonstrated importance of EVs in the modulation of the immune response of infection and their altered concentration in circulation, EVs present themselves as promising candidates for biomarkers of disease progression in Chagas disease.

## Figures and Tables

**Table 1:** Patients' and controls' baseline and clinical characteristics. Chronic Chagas disease patients (CCD; *n* = 40) and controls (CTRL; *n* = 30).

		Chronic Chagas disease (CCD)	Healthy controls (CTRL)
Sex	Male	17	10
	Female	23	20

Age	<20	0	1
	20-39	1	27
	40-59	18	2
	60-80	20	0
	>80	1	0

Clinical stage of cardiac burden	Indeterminate	10	—
	ECG alteration	15	—
	ECG alteration + ventricular dysfunction	15	—

NYHA functional classification	I	28	—
	II	8	—
	IV	4	—
		160	60

ECG: electrocardiogram; NYHA: New York Heart Association.

## Data Availability

Data are available on request.

## Supplementary Materials

Supplementary Materials Supplementary Table 1: full clinical data from patients and controls.
