# Supplementary material for: New Biomarker in Chagas Disease: Extracellular Vesicles Isolated from Peripheral Blood in Chronic Chagas Disease Patients Modulate the Human Immune Response
Source: J Immunol Res. 2021 Jan 11;2021:6650670. doi: 10.1155/2021/6650670 (PMC7815414; doi:10.1155/2021/6650670)
Supplement: Supplementary Materials — Supplementary Table 1: full clinical data from patients and controls. [file 6650670.f1.zip › SUPPLEMENTARY DESCRIPTIO2.docx]

**SUPPLEMENTARY DESCRIPTION:**

**Supplementary Table 1.** Full clinical data from patients and controls.
