# Supplementary material for: New Biomarker in Chagas Disease: Extracellular Vesicles Isolated from Peripheral Blood in Chronic Chagas Disease Patients Modulate the Human Immune Response
Source: J Immunol Res. 2021 Jan 11;2021:6650670. doi: 10.1155/2021/6650670 (PMC7815414; doi:10.1155/2021/6650670)
Supplement: Supplementary Materials — Supplementary Table 1: full clinical data from patients and controls. [file 6650670.f1.zip › Supplementary table_Madeira et al. 2020 (1).pdf]

Number ID (*controls)	Sex	Age	Infection in the last 15 days	Antiinflammatories in the last 15 days	Clinical form/ Cardiac Burden
**1**	F	56	N	N	indeterminate
**2**	M	55	N	N	electrocardiogram alteration and ventricular dysfunction
**3**	M	70	N	N	electrocardiogram alteration
**4**	M	55	N	N	indeterminate
**5**	F	73	N	N	indeterminate
**6**	M	42	N	N	electrocardiogram alteration and ventricular dysfunction
**7**	M	62	N	N	indeterminate
**8**	F	57	N	N	electrocardiogram alteration and ventricular dysfunction
**9**	F	59	N	N	electrocardiogram alteration and ventricular dysfunction
**10**	F	72	N	N	electrocardiogram alteration
**11**	F	71	N	N	electrocardiogram alteration and ventricular dysfunction
**12**	F	55	Y (cold)	N	electrocardiogram alteration
**13**	F	61	N	N	electrocardiogram alteration
**14**	M	53	N	N	electrocardiogram alteration and ventricular dysfunction
**15**	M	71	N	N	electrocardiogram alteration and ventricular dysfunction
**16**	F	79	N	N	indeterminate
**17**	F	57	N	N	electrocardiogram alteration and ventricular dysfunction
**18**	M	60	N	N	electrocardiogram alteration
**19**	M	46	N	N	electrocardiogram alteration and ventricular dysfunction

**20**	M	57	N	N	electrocardiogram alteration and ventricular dysfunction
**21**	F	62	N	N	electrocardiogram alteration
**22**	F	58	N	N	electrocardiogram alteration
**23**	M	43	N	N	electrocardiogram alteration and ventricular dysfunction
**24**	F	67	N	N	electrocardiogram alteration
**25**	M	70	N	N	electrocardiogram alteration
**27**	F	58	N	N	electrocardiogram alteration
**28**	F	72	N	N	indeterminate
**29**	F	75	N	N	electrocardiogram alteration
**30**	M	67	N	N	electrocardiogram alteration
**31**	F	63	N	N	electrocardiogram alteration
**32**	M	69	N	N	indeterminate
**33**	F	45	N	N	electrocardiogram alteration
**34**	M	63	N	N	electrocardiogram alteration and ventricular dysfunction
**35**	M	81	N	N	indeterminate
**36**	F	46	N	N	electrocardiogram alteration and ventricular dysfunction
**37**	F	51	N	N	electrocardiogram alteration and ventricular dysfunction
**38**	F	30	N	N	electrocardiogram alteration and ventricular dysfunction
**39**	F	49	N	N	indeterminate
**40**	F	76	N	N	indeterminate
**41**	M	65	N	N	electrocardiogram alteration
1*	F	30	N	N	-----
2*	F	26	N	N	-----
3*	M	30	N	N	-----
4*	F	50	N	N	-----
5*	F	20	N	N	-----
6*	M	22	N	N	-----
7*	F	39	N	N	-----

8*	F	26	N	N	-----
9*	F	25	N	N	-----
10*	F	36	N	N	-----
11*	F	32	N	N	-----
12*	M	32	N	N	-----
13*	M	26	Y (cold)	N	-----
14*	M	25	N	N	-----
15*	F	25	N	N	-----
16*	F	41	N	N	-----
17*	F	26	N	N	-----
18*	M	37	N	N	-----
19*	F	29	N	N	-----
20*	M	24	N	N	-----
21*	F	24	N	N	-----
22*	F	23	N	N	-----
23*	M	19	N	N	-----
24*	M	24	N	N	-----
25*	F	28	N	N	-----
26*	F	21	N	N	-----
27*	M	21	N	N	-----
28*	F	21	N	N	-----
29*	F	24	N	N	-----
30*	F	26	N	N	-----

NYHA Functional Class	Ejection Fraction	Heart Rhythm	EVs Mean Concentration (particles/mL)	EVs Mean Size (nm)
II	0.65	NL	1,51E+09	188,6
IV	0.31	left atrial overload, complete right bundle branch block, left anterior fascicular block, premature ventricular contraction	6,80E+08	179,5
I	0.71	atrial fibrillation	1,25E+09	156,5
I	0.66	NL	8,39E+08	156,2
I	0.68	NL	3,22E+08	208,1
II	0.4	sinus bradycardia, left anterior fascicular block, complete right bundle branch block, non-specific ventricular repolarization alteration	3,84E+08	266,2
I	0.6	final conduction delay	2,08E+08	121,6
II	0.2	non-specific intraventricular conduction delay, non-specific ventricular repolarization alteration, left anterior fascicular block	4,54E+08	163,6
IV	0.3	pacemaker	3,63E+08	236,1
I	0.5	left anterior fascicular block, complete right bundle branch block	3,28E+08	225,6
IV	0.2	sinus bradycardia, left anterior fascicular block, complete right bundle branch block	1,45E+08	212,6
I	0.41	left anterior fascicular block, complete right bundle branch block, non-specific ventricular repolarization alteration	8,10E+08	196,8
II	0.7	left anterior fascicular block, complete right bundle branch block, anteroseptal electrical inactivity, first degree atrioventricular block	2,65E+08	264,2
I	0.3	pacemaker	2,01E+08	110,5
II	0.3	complete right bundle branch block, left anterior fascicular block, premature ventricular contraction	7,90E+08	177,5
I	0.6	NL	1,61E+09	241,8
I	0,39	left anterior fascicular block, complete right bundle branch block, non-specific ventricular repolarization alteration	1,24E+09	214,2
I	0,58	non-specific ventricular repolarization alteration	3,09E+08	169,2
I	0,25	left ventricular overload, complete right bundle branch block, left anterior fascicular block	5,76E+08	212,4

IV	0,22	final conduction delay, left atrial overload, premature ventricular contraction, inferior electrical inactivity, non-specific ventricular repolarization alteration	1,11E+09	186,5
I	0,57	left anterior fascicular block, final conduction delay, left ventricular overload	3,55E+08	230,6
I	0,60	non-specific ventricular repolarization alteration	1,44E+08	261,3
I	0,20	final conduction delay, non-specific ventricular repolarization alteration	1,35E+08	303,3
I	0,57	first degree atrioventricular block, septal force diminishment, non-specific ventricular repolarization alteration	1,70E+08	189,1
I	0,62	complete right bundle branch block, inferior electrical inactivity	2,21E+08	275,7
I	0,61	premature ventricular contraction, BIG	6,14E+08	257,2
I	0,65	NL	1,72E+06	72,7
II	0,66	isolated premature ventricular contraction	1,90E+08	263,1
I	0,66	atrial fibrilation, left anterior fascicular block	1,82E+08	199,7
I	0,66	left anterior fascicular block, non-specific ventricular repolarization alteration	6,23E+08	137,8
I	0,81	NL	1,87E+08	216,6
I	0,69	sinus ectopic rhythm	3,41E+07	167,6
I	0,45	non-specific ventricular repolarization alteration	1,37E+08	279
I	0,68	NL	4,07E+08	277,9
II	0,35	premature ventricular contraction, non-specific ventricular repolarization alteration	5,45E+08	336,4
I	0,33	pacemaker	5,11E+07	190,6
I	0,40	premature ventricular contraction, left anterior fascicular block, final conduction delay	2,93E+08	176,6
I	0,64	NL	4,03E+08	163,9
II	0,62	NL	6,25E+08	202,4
I	0,62	left anterior fascicular block, complete right bundle branch block	2,58E+08	166,6
-----	-----	-----	1,27E+09	144,2
-----	-----	-----	7,71E+08	161
-----	-----	-----	6,02E+08	164
-----	-----	-----	1,04E+09	173,2
-----	-----	-----	9,56E+08	184,1
-----	-----	-----	4,27E+08	100,9
-----	-----	-----	1,10E+09	195,8

-----	-----	-----	6,95E+08	191,6
-----	-----	-----	6,18E+08	199,6
-----	-----	-----	2,18E+07	361,1
-----	-----	-----	1,49E+08	226,8
-----	-----	-----	2,42E+08	237,6
-----	-----	-----	6,03E+07	282,6
-----	-----	-----	3,87E+07	270,5
-----	-----	-----	3,05E+07	284,7
-----	-----	-----	8,58E+07	179
-----	-----	-----	1,47E+08	147,4
-----	-----	-----	1,70E+08	132
-----	-----	-----	5,02E+08	170,1
-----	-----	-----	8,18E+08	267,4
-----	-----	-----	6,95E+08	249,4
-----	-----	-----	4,36E+08	228,8
-----	-----	-----	1,09E+09	280,2
-----	-----	-----	1,12E+09	275,6
-----	-----	-----	4,76E+08	183,9
-----	-----	-----	5,67E+08	192,9
-----	-----	-----	1,54E+08	176,4
-----	-----	-----	4,03E+08	129,5
-----	-----	-----	6,28E+08	157,7
-----	-----	-----	3,23E+08	167,4

EVs D10 (nm)	EVs D50 (nm)	EVs D90 (nm)	THP-1 IFN-γ (pg/mL)	THP-1 IL-17 (pg/mL)
109,2	173,2	280,3	6,247694	7,552713
62	173,7	312,6	6,580625	7,552713
46,4	119,1	332,3	6,25507	7,470708
85,7	135,8	249	6,112791	7,373477
128,3	190,5	292,6	6,366765	7,487038
127,5	251,6	400,5	6,116399	6,860889
45,4	113,6	216,6	6,25507	7,022552
65	159,1	293,2	5,77961	7,135899
78,1	222,9	399,6	6,25507	7,53624
76,7	226,8	420,1	6,269849	7,487038
148,3	199,8	281	6,170766	7,400358
66	176,1	352,2	6,247694	7,448991
123,3	240,1	432,1	5,963185	7,352043
31,6	87,5	209,3	6,295795	7,427337
113,6	157,7	278,1	6,15259	7,378845
141,8	215,9	394,1	6,252964	6,376693
143	192,1	305	6,244285	6,362175
121,3	146,5	224,4	6,455936	6,391243
113,9	191,1	351,9	6,23129	6,383964

122,2	179,4	277	5,829415	6,362175
129,3	207	423	6,252964	6,208187
164,4	258	363,6	6,218321	6,265045
161,8	272,4	461,4	6,389036	6,329632
101,4	176,2	316,8	6,40236	6,380327
164,2	223,6	426,7	6,982955	6,416786
137,4	230,4	423,2	6,810546	6,409478
67,1	72,2	77,8	5,993613	6,413131
166,1	232,7	376,5	5,685486	6,394886
148	208,6	238,9	6,491902	6,424103
78,4	113,1	240,8	5,809209	6,387602
133,6	204,7	338,7	6,455936	6,383964
56,7	137,2	310	6,455936	6,37306
133	282,2	427,4	6,546227	6,318822
154,7	264	427,4	6,791654	6,365802
169,9	334,8	523	6,4425	6,380327
123	170,4	306,3	6,261654	6,409478
115,5	148,7	279,3	6,537142	6,416786
106,6	135,6	265,3	6,188167	6,402178
129,3	180,1	330,3	6,287799	6,402178
101	137,7	275,9	6,054232	6,835234
97,2	129,4	205,6	5,949129	7,384218
114,6	133,6	242,8	6,13085	7,362752
112,3	148,7	239,2	6,087599	7,389594
118,3	153,4	243,5	6,037525	7,378845
95,5	161,5	292,1	6,214608	7,394974
23,2	85,5	181,5	6,167127	7,389594
129,7	169,8	302,8	5,994932	7,336008

119,5	168,7	285,5	5,942114	7,368113
128,4	180,4	278,9	5,64664	6,714969
333,4	362	383,1	5,807156	6,618542
155,7	202,6	343,5	6,068008	6,794908
85,1	223,6	400,5	6,2122	6,78598
197,2	237,5	440,4	6,231054	6,732652
139,5	252,2	410	6,193404	6,653445
131,7	230	541,6	6,054232	6,675353
66	163,8	334,8	6,169988	6,803849
66,2	135	215,8	6,235776	6,87128
57,8	120,7	231	6,179344	6,794908
113,7	152,8	234,8	6,132708	6,799377
156,6	219,5	461,2	6,174664	6,821766
158,3	208,7	406,6	5,99036	6,768157
136,3	200	358,4	6,264185	6,781519
156,9	231,8	454,5	6,031343	6,772608
160,2	231,1	469,4	6,00854	6,925706
106,2	174,7	279,6	6,142007	6,799377
120,3	163,7	317,2	5,846885	6,723805
103,1	163,1	269,6	6,114152	6,893905
80,8	114,5	211	5,967712	6,889374
97,1	135,5	240,9	6,013094	6,907516
110,8	154,1	233	6,297492	6,934819
